# Efficacy and safety of PD-1 inhibitors in combination with chemotherapy as first-line treatment for HER2-negative advanced gastric or gastroesophageal junction cancer across subgroups: A comprehensive systematic review and meta-analysis

**DOI:** 10.1097/MD.0000000000041751

**Published:** 2025-08-15

**Authors:** Muhetaibaier Hairoula, Yu Wei, Kalima Muhetaer, Xiaoli Ma, Leiyu Cao, Yan Gao, Chengcheng Qu, Wen Yi, Li Zhang

**Affiliations:** a Cadre Healthcare Center, The First Affiliated Hospital of Xinjiang Medical University, Urumqi, China.

**Keywords:** efficacy, first-line treatment, gastroesophageal junction cancer, HER2-negative advanced gastric cancer, PD-1 inhibitor, safety

## Abstract

**Background:**

The advent of immune checkpoint inhibitors has introduced innovative therapeutic paradigms for the management of human epidermal growth factor receptor 2 (HER2)-negative advanced gastric or gastroesophageal junction cancer (GC/GEJC). However, the efficacy and safety of programmed cell death protein 1 (PD-1) inhibitors combined with chemotherapy versus chemotherapy alone in patients with HER2-negative advanced GC/GEJC remain contentious. The comparability among different subgroups is not fully understood, necessitating the identification of optimal patient demographics and the exploration of potential biomarkers.

**Methods:**

This study identified 6 Phase III randomized controlled trials evaluating the first-line treatment of HER2-negative GC/GEJC with PD-1 inhibitors in combination with chemotherapy. The primary endpoints include overall survival (OS), progression-free survival (PFS), and objective response rate (ORR), assessed using hazard ratios (HR), relative risk, and their respective 95% confidence intervals (CI). Secondary outcomes are treatment-related adverse events and immune-related adverse events. Prespecified subgroups encompass microsatellite instability, programmed death-ligand 1 (PD-L1) combined positive score (CPS), age, gender, previous surgery, primary location, liver metastases, Eastern Cooperative Oncology Group performance status score, histological subtype, chemotherapy regimen, race, and PD-L1 expression in tumor cells.

**Results:**

Incorporating data from 6 randomized controlled trials, this analysis included 6294 adult patients with HER2-negative advanced GC/GEJC. The combined PD-1 inhibitor and chemotherapy regimen significantly improved OS (*HR* = 0.79, 95% CI [0.75, 0.84], *P* < .00001) and PFS (*HR* = 0.75, 95% CI [0.70, 0.80], *P* < .00001), along with enhancing the ORR (relative risk = 1.22, 95% CI [1.15, 1.29], *P* < .00001). Subgroup analyses revealed benefits in OS, PFS, and ORR for patients with CPS ≥ 1, CPS ≥ 5, and CPS ≥ 10 when treated with first-line PD-1 inhibitors and chemotherapy, with higher PD-L1 expression levels correlating with greater efficacy. Furthermore, patients with high microsatellite instability exhibited a more pronounced extension in OS (*HR* = 0.35, 95% CI [0.21, 0.59], *P* < .0001). However, factors such as age, gender, previous surgery, primary location, liver metastases, Eastern Cooperative Oncology Group performance status score, histological subtype, chemotherapy regimen, race, and PD-L1 expression in tumor cells were not predictive of an OS benefit from PD-1 inhibitors combined with chemotherapy over chemotherapy alone. Regarding safety, the PD-1 inhibitor and chemotherapy combination led to a higher incidence of immune-mediated adverse events, though there was no significant difference in adverse events leading to death.

**Conclusion:**

First-line treatment with PD-1 inhibitors combined with chemotherapy surpasses chemotherapy alone in efficacy for patients with HER2-negative advanced GC/GEJC, particularly in those with CPS ≥ 10 or high microsatellite instability, with tolerable adverse events.

## 1. Introduction

Gastric or gastroesophageal junction cancer (GC/GEJC) are characterized by high incidence rates, high mortality rates, and poor prognoses.^[1]^ Annually, there are over 1 million new cases of GC worldwide, ranking it as the fifth most common malignancy globally.^[2]^ Human epidermal growth factor receptor 2 (HER2)-negative GC is a subtype with high incidence, usually without obvious symptoms in the early stage, and most of the patients have already progressed to the advanced stage by the time of definitive diagnosis, as well as with poor efficacy and prognosis.^[3]^ In recent years, immune checkpoint inhibitors (ICIs), including nivolumab, pembrolizumab, sintilimab, and tislelizumab, have made significant breakthroughs in the first-line treatment of advanced GC. However, the efficacy and safety of programmed cell death protein (PD-1) inhibitors in combination with chemotherapy in HER2-negative advanced GC/GEJC patients remain controversial. For example, the phase III clinical trial KEYNOTE-859 study showed that nivolumab combination chemotherapy significantly prolonged survival in patients with HER2-negative advanced GC/GEJC compared to chemotherapy alone.^[4]^ The results of the ATTRACTION-4 trial, on the other hand, demonstrated that the nivolumab combination chemotherapy strategy did not improve overall survival (OS) compared to chemotherapy alone.^[5]^ The KEYNOTE-062 trial reported that pembrolizumab in combination with chemotherapy was not superior to chemotherapy in prolonging OS and progression-free survival (PFS) in patients with HER2-negative advanced GC/GEJC with combined positive score (CPS) ≥ 1.^[6]^ Therefore, it is essential to identify the optimal patient population while exploring whether HER2-negative advanced GC/GEJC patients benefit from PD-1 inhibitor combination chemotherapy regimens. Furthermore, programmed death-ligand 1 (PD-L1) expression is considered a prognostic biomarker and is expressed in 30% to 65% of invasive GCs.^[7,8]^ However, the CPS threshold that allows for optimal patient benefit has not been clarified. Additionally, microsatellite instability (MSI) has shown some potential in predicting the efficacy of ICIs in solid tumors,^[9,10]^ but its role in GC/GEJC remains controversial. As for this reasons, our study also aims to find prognostic markers for effective assessment of efficacy for this group of patients, in anticipation of providing value for their clinical diagnosis and treatment.

## 2. Materials and methods

### 2.1. Search strategy

A comprehensive search was conducted across PubMed, Web of Science, Embase, and the Cochrane controlled trials register, as well as abstracts from the American Society of Clinical Oncology (ASCO) and the European Society for Medical Oncology (ESMO) meetings, for randomized controlled trials (RCTs) concerning the use of PD-1 inhibitors in combination with chemotherapy as first-line treatment in patients with HER2-negative advanced GC/GEJC. Search terms included “advanced GC, gastroesophageal-junction cancer, PD-1 inhibitor, immunotherapy, pembrolizumab, nivolumab, sintilimab, tislelizumab, and chemotherapy” with a language restriction to English. The systematic search spanned from March 2019 to March 2024 for RCTs and conference abstracts relevant to this study, evaluating the latest literature. Additionally, conference proceedings from the past 5 years from the ASCO, ESMO, ASCO Gastrointestinal, and ESMO Gastrointestinal meetings were explored, and citation lists were meticulously reviewed to identify and supplement unpublished data relevant to the research.

### 2.2. Inclusion and exclusion criteria

The literature eligible for inclusion met the following criteria: multicenter, randomized, controlled, Phase III clinical trials. Trials involving patients with unresectable, metastatic, or locally advanced HER2/ERBB2-negative GC/GEJC. Trials including patients aged ≥18 years. Trials where the treatment regimen was designated as first-line therapy. Trials that provided sufficient tumor tissue for PD-L1 assessment and conducted stratified analyses based on PD-L1 CPS. Trials where the patients had an Eastern Cooperative Oncology Group (ECOG) performance status score of 0 or 1. Trials that allowed disease assessment based on the solid tumor response criteria (RECIST, version 1.1). Trials that reported at least one of the following outcome measures: OS, PFS, objective response rate (ORR), treatment-related adverse event (TRAE), immune-related adverse events (irAEs) and provided relevant data including hazard ratios (HR), relative risk (RR), number of clinical responses, total number of participants per group, and data pertinent to subgroups. Clinical trials comparing PD-1 inhibitor combined with chemotherapy against monotherapy chemotherapy or chemotherapy combined with placebo.

Exclusion criteria were as follows: Phase I or II trials or non-randomized controlled studies. Studies not involving GC or patients with early-stage GC/GEJC. Trials with HER2/ERBB2 positive patients. Studies focusing on first-line maintenance treatment, second-line, or third-line treatments. Studies lacking specific data on survival and clinical efficacy. Trials not comparing PD-1 inhibitor combined with chemotherapy against monotherapy chemotherapy or chemotherapy combined with placebo. Trials with unclear clinical outcomes.

### 2.3. Data extraction and quality assessment

All data utilized in this study were derived from publications and pertinent online supplemental materials. Literature screening and data extraction were collaboratively conducted by 2 researchers (MH and YW), with cross-verification to ensure accuracy. In instances of disagreement, a third researcher (LZ) intervened to achieve consensus. For trials with 3 arms, only data pertinent to the inclusion criteria and relevant to the study’s objectives were extracted. Key data points included the trial name, publication year, NCT number, interventions for both groups, sample size, whether the treatment was first-line, target mechanism, CPS threshold, HR with 95% confidence intervals (CI), and event counts, among others. A total of 6294 adult patients with HER2-negative advanced GC/GEJC were included in the trials, with 3026 receiving PD-1 inhibitor combined with first-line chemotherapy and 3012 in the control group receiving chemotherapy or chemotherapy combined with placebo. All 6 RCTs reported the primary outcome measures and related data required for this study, with additional characteristics detailed in Table 1. The quality of the included RCTs was assessed using a modified Jaded scale, with scores ranging from 0 to 3 indicating low quality and scores from 4 to 7 indicating high quality, as presented in Table 2.

**Table 1:** Basic characteristics of included studies.

Trial	RATIONALE 305	KEYNOTE-859	ORIENT-16	ATTRACTION-4	CheckMate 649	KEYNOTE-062
Year	2023	2023	2023	2022	2021	2020
Phase	III	III	III	III	III	III
NCT identifier	NCT03777657	NCT03675737	NCT03745170	NCT02746796	NCT02872116	NCT02494583
Stage	Locally advanced or metastatic	Locally advanced or metastatic	Locally advanced or metastatic	Advanced or recurrent	Advanced	Locally advanced or metastatic
Type of cancer	GC/GEJC	GC/GEJC	GC/GEJC	GC/GEJC	GC/GEJC/EAC	GC/GEJC
HER2/ERBB2	Negative	Negative	Negative	Negative	Negative	Negative
Target	PD-1	PD-1	PD-1	PD-1	PD-1	PD-1
Line of therapy	First	First	First	First	First	First
Total number	997	1579	650	724	1581	763
Randomization	1:1	1:1	1:1	1:1	1:1	1:1:1
Study arm (n)	Tislelizumab + chemotherapy (501)	Pembrolizumab + chemotherapy (790)	Sintilimab + chemotherapy (327)	Nivolumab + chemotherapy (362)	Nivolumab + chemotherapy (789)	Pembrolizumab + chemotherapy (257)
Control arm (n)	Placebo + chemotherapy (496)	Placebo + chemotherapy (789)	Chemotherapy alone (323)	Placebo + chemotherapy (362)	Chemotherapy alone (792)	Chemotherapy alone (250)
Outcome	OS, PFS, ORR, TRAE	OS, PFS, ORR, TRAE, irAEs	OS, PFS, ORR, TRAE, irAEs	OS, PFS, ORR, TRAE	OS, PFS, ORR, TRAE	OS, PFS, ORR, TRAE
CPS cutoff	NA	1,10	5	NA	1,5	1,10

CPS = combined positive score, EAC = esophageal adenocarcinoma , GC/GEJC = gastric or gastroesophageal junction cancer, HER2 = human epidermal growth factor receptor 2, irAEs = immune-related adverse events, NA = not available, ORR = objective response rate, OS = overall survival, PD-1 = programmed cell death protein 1, PFS = progression-free survival, TRAE = treatment-related adverse event.

**Table 2:** Modified Jaded scores of included studies.

Study	Random sequence generation	Allocation concealment	Masking	Withdrawal/lost visit	Score	Level
RATIONALE 305*	1	1	1	0	3	Low
KEYNOTE-859	2	2	2	1	7	High
ORIENT-16	2	1	1	1	5	High
ATTRACTION-4	2	2	2	1	7	High
CheckMate649	2	2	2	1	7	High
KEYNOTE-062	2	1	1	1	5	High

* As the RATIONALE 305 has only been presented in the form of abstracts/oral presentations and the full manuscripts have not yet been published, data for quality assessment are limited.

### 2.4. Statistical analysis

**Figure 1.**
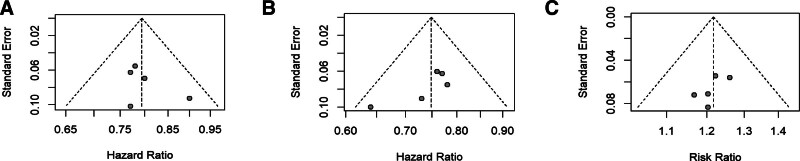
Assessment of bias risk. (A) OS, (B) PFS, (C) ORR. ORR = objective response rate, OS = overall survival, PFS = progression-free survival.

Meta-analysis was performed using R 4.3.1 software. In this study, generic inverse variance was employed to calculate OS and PFS, while dichotomous analysis was used for ORR, TRAE, and irAEs. Heterogeneity testing was conducted for each analysis using the chi-squared test, with *I*^*2*^ ≤ 50% or *P* ≥ .05 indicating good homogeneity among the studies, allowing for the application of a fixed-effects model for pooling and analysis. Conversely, *I*^*2*^ > 50% or *P* < .05 indicated heterogeneity among the studies, warranting random-effects model analysis and sensitivity analysis to explore potential sources of heterogeneity. A 2-tailed *P*-value < 0.05 was considered statistically significant for differences. Using R 4.3.1 software, Egger’s regression method was utilized to assess publication bias for OS (*P* = .4876), PFS (*P* = .1172), and ORR (*P* = .3201), with results indicating no significant publication bias across the evaluated outcomes, as presented in Figure 1.

## 3. Results

### 3.1. Study selection and characteristics

**Figure 2.**
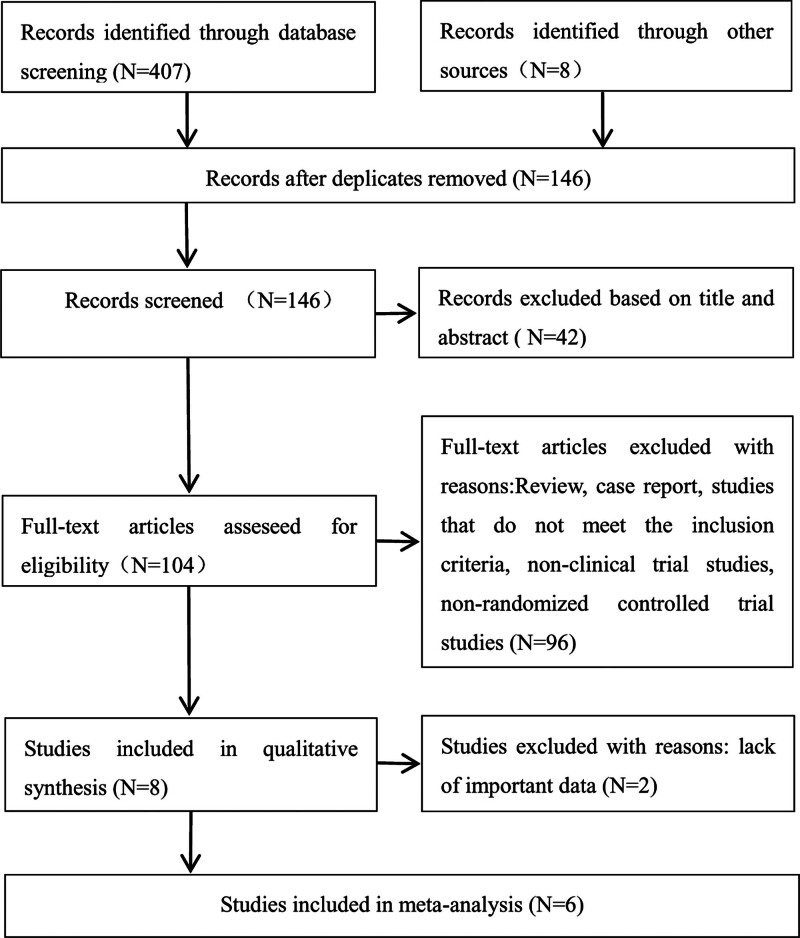
Flow diagram.

An initial screening identified 407 publications, with an additional 8 sources from other origins. Following the removal of duplicates, 146 publications remained. A preliminary screening of titles and abstracts further narrowed the selection to 104 publications. After excluding reviews, case reports, studies not meeting inclusion criteria, nonclinical trials, and non-RCT literature, 8 publications remained. Two of these did not contain the critical data required for our study, thus leaving 6 for final inclusion (Fig. 2). The basic characteristics of the clinical trials included are detailed in Table 1. The study ultimately included 6 Phase III clinical trials conducted over the past 5 years,^[4–6,11–13]^ focusing on first-line treatments for advanced GC/GEJC, all involving PD-1 inhibitors for HER2/ERBB2-negative patients. The RATIONALE 305 trial, a global, double-blind, Phase III study, has only been reported in abstract/oral presentation form, and since the full text has not been published, data for quality assessment is limited,^[11]^ only preliminary analysis results for the intended population are included herein. The KEYNOTE-062 trial included 3 arms: chemotherapy alone, pembrolizumab monotherapy, and chemotherapy combined with pembrolizumab.^[6]^ To compare the efficacy of PD-1 inhibitors in combination with chemotherapy versus chemotherapy alone, only data from the combination therapy group and data from the chemotherapy group of patients with CPS ≥ 1 and CPS ≥ 10 were included in this study. The CheckMate-649 trial initially recruited patients across 3 groups in a 1:1:1 ratio: chemotherapy, nivolumab combined with chemotherapy, and nivolumab combined with ipilimumab.^[12]^ After recruitment for the nivolumab combined with ipilimumab group ended, the trial proceeded with a 1:1 ratio, reporting only the results for patients randomly assigned to nivolumab combined with chemotherapy versus chemotherapy alone. Results for nivolumab combined with ipilimumab versus chemotherapy alone remain undisclosed, thus this study extracted data for the reported nivolumab combined with chemotherapy and chemotherapy alone groups, including all randomized participants and those with CPS ≥ 1 and CPS ≥ 5. The ATTRACTION-4 trial recruited patients in a 1:1 ratio for nivolumab combined with chemotherapy and chemotherapy combined with placebo groups, from which we extracted data on efficacy, survival, and drug-related adverse events for all randomized participants.^[5]^ The ORIENT-16 trial is the first Phase III study to report the efficacy and safety of sintilimab combined with chemotherapy versus placebo combined with chemotherapy as first-line treatment for advanced GC/GEJC, with the latest results released in December 2023 by Professor Xu Jianming’s team, including the full text and supplemental materials.^[13]^ Data on efficacy, survival, drug-related adverse events, and irAEs were extracted for all randomized participants and those with CPS ≥ 5 from the ORIENT-16 trial. The KEYNOTE-859 is a prospective, randomized, double-blind, placebo-controlled, international, pivotal Phase III clinical trial, with the latest findings published in December 2023 by the team led by Sun Young Rha, including full text and supplemental materials.^[4]^ Data on efficacy, survival, drug-related adverse events, and irAEs for all randomized participants and those with CPS ≥ 1 and CPS ≥ 10 were extracted. The 6 studies included are multicenter, randomized, controlled, Phase III clinical trials, and an assessment of bias risk indicated that all included studies were of acceptable quality.

## 4. Efficacy

**Figure 3.**
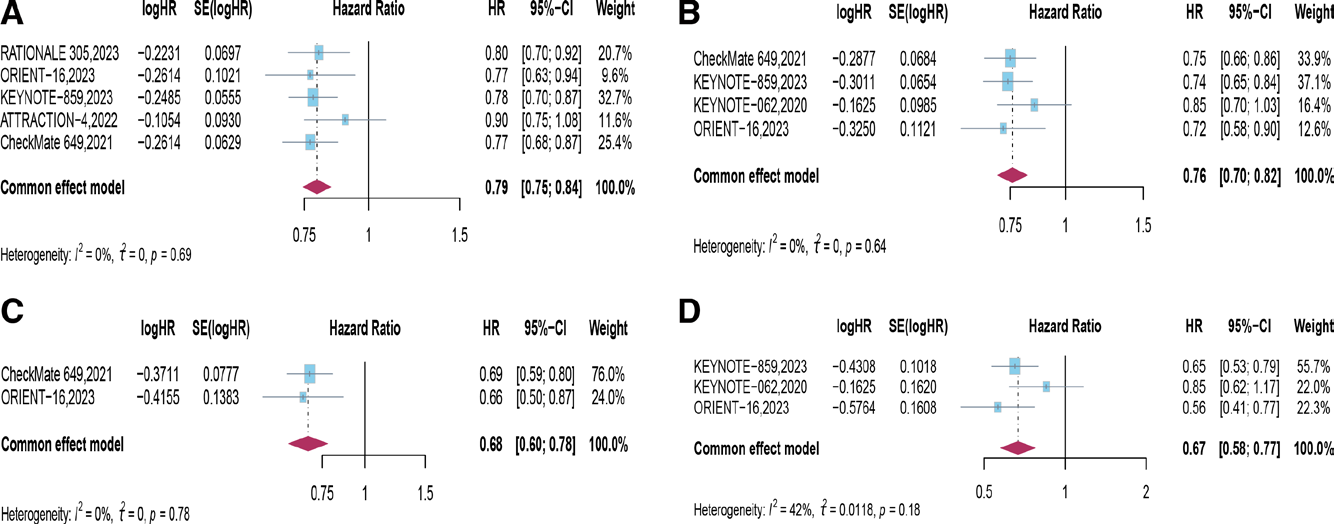
Forest plots of hazard ratios (HRs) comparing overall survival (OS) between PD-1 inhibitor combined with chemotherapy and chemotherapy based on (A) intention-to-treat (ITT), (B) PD-L1 combined positive score (CPS) ≥ 1, (C) CPS ≥ 5, and (D) CPS ≥ 10. CI = confidence intervals, CPS = combined positive score, HRs = hazard ratios, PD-1 = programmed cell death protein 1, PD-L1 = programmed death-ligand 1, OS = overall survival, ITT = intention-to-treat.

Five trials examined the long-term efficacy of PD-1 inhibitor combined with chemotherapy in the intended population, revealing a significant extension in patients’ OS with the first-line treatment regimen (*HR* = 0.79, 95% CI [0.75, 0.84], *P* < .00001; Fig. 3A). Subgroup analysis based on PD-L1 expression in this study demonstrated that when patients had CPS ≥ 1 (*HR* = 0.76, 95% CI [0.70, 0.82], *P* < .00001), CPS ≥ 5 (*HR* = 0.68, 95% CI [0.60, 0.78], *P* < .00001), and CPS ≥ 10 (*HR* = 0.67, 95% CI [0.58, 0.77], *P* < .00001), PD-1 inhibitor combined with chemotherapy as first-line treatment for HER2-negative advanced GC/GEJC extended OS significantly. Moreover, the higher the CPS score, the more pronounced the benefits observed (Fig. 3B–D, Table 3).

**Table 3:** Pooled hazard ratio for clinical efficacy and safety.

			Heterogeneity		Effect size	*P*-value	Model
			*I*^*2*^ (%)	*P*			
Efficacy	OS	ITT	0	.69	HR 0.79 [0.75,0.84]	<.00001	Common
		CPS ≥ 1	0	.64	HR 0.76 [0.70,0.82]	<.00001	Common
		CPS ≥ 5	0	.78	HR 0.68 [0.60,0.78]	<.00001	Common
		CPS ≥ 10	42	.18	HR 0.67 [0.58,0.77]	<.00001	Common
	PFS	ITT	0	.54	HR 0.75 [0.79,0.80]	<.00001	Common
		CPS ≥ 1	0	.41	HR 0.75 [0.69,0.82]	<.00001	Common
		CPS ≥ 5	0	.54	HR 0.67 [0.59,0.77]	<.00001	Common
		CPS ≥ 10	0	.39	HR 0.65 [0.55,0.77]	<.00001	Common
	ORR	ITT	0	.94	RR 1.22 [1.15,1.29]	<.00001	Common
		CPS ≥ 1	0	.71	RR 1.27 [1.17,1.37]	<.00001	Common
		CPS ≥ 5	0	.83	RR 1.31 [1.17,1.46]	<.00001	Common
		CPS ≥ 10	0	.95	RR 1.40 [1.21,1.63]	<.00001	Common
Safety	TRAE	Any	71	.01	RR 1.02 [1.00,1.05]	.656	Random
		Grade ≥ 3	55	.06	RR 1.18 [1.10,1.28]	<.0001	Random
		Discontinuation	19	.29	RR 1.49 [1.34,1.67]	<.0001	Common
		Serious AEs	65	.04	RR 1.47 [1.19,1.80]	.0003	Random
		Led to death	64	.03	RR 1.64 [0.66,4.04]	.2837	Random
	irAEs	Any	90	<.01	RR 2.19 [1.24,3.87]	.007	Random
		Grade ≥ 3	0	.34	RR 4.01 [2.59,6.21]	<.001	Common

AE = adverse event, CPS = combined positive score, HER2 = human epidermal growth factor receptor 2, HR = hazard ratios, irAEs = immune-related adverse events, ITT = intention-to-treat, ORR = objective response rate, OS = overall survival, PFS = progression-free survival, RR = relative risk, TRAE = treatment-related adverse event.

**Figure 4.**
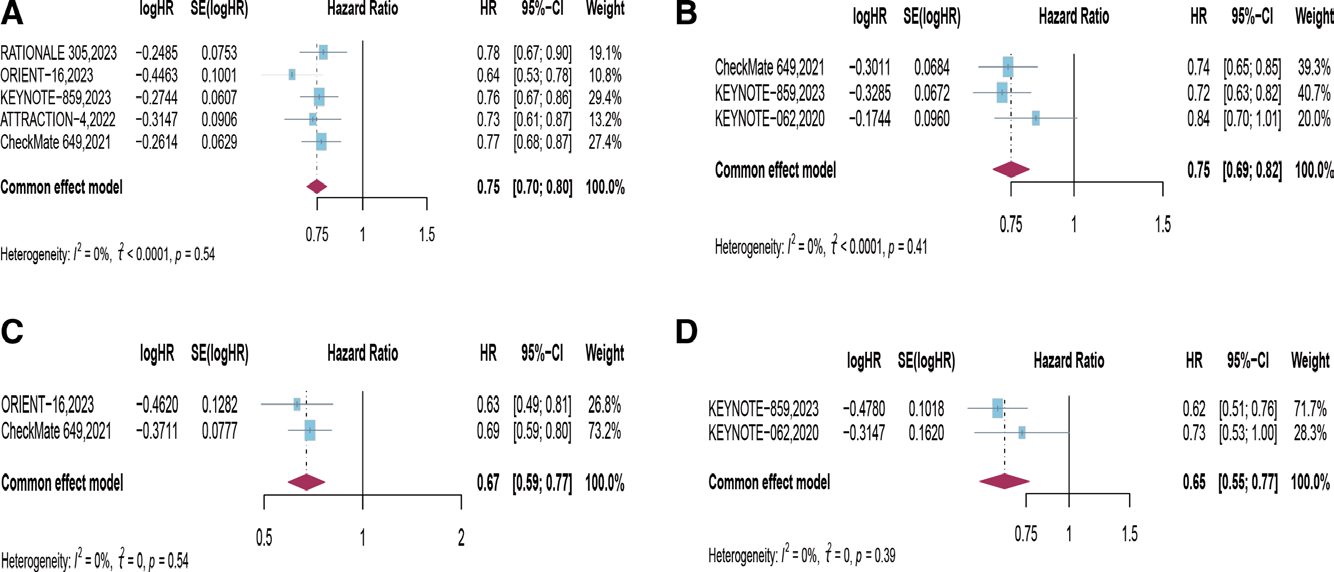
Forest plots of hazard ratios (HRs) comparing progression-free survival (PFS) between PD-1 inhibitor combined with chemotherapy and chemotherapy based on (A) Intention-to-Treat (ITT), (B) PD-L1 combined positive score (CPS) ≥ 1, (C) CPS ≥ 5, and (D) CPS ≥ 10. CI = confidence intervals, CPS = combined positive score, HRs = hazard ratios, PD-1 = programmed cell death protein 1, PD-L1 = programmed death-ligand 1, PFS = progression-free survival, ITT = intention-to-treat.

Four trials investigated the long-term efficacy of PD-1 inhibitor combined with chemotherapy in the intended population, demonstrating that the first-line treatment regimen of PD-1 inhibitor combined with chemotherapy significantly prolonged patients’ PFS (*HR* = 0.75, 95% CI [0.70, 0.80], *P* < .00001; Fig. 4A). Subgroup analysis based on PD-L1 expression in this study revealed that when CPS ≥ 1 (*HR* = 0.75, 95% CI [0.69, 0.82], *P* < .00001), CPS ≥ 5 (*HR* = 0.67, 95% CI [0.59, 0.77], *P* < .00001), and CPS ≥ 10 (*HR* = 0.65, 95% CI [0.55, 0.77], *P* < .00001), patients still benefited in terms of PFS, with greater benefits observed with higher CPS scores (Fig. 4B–D, Table 3).

**Figure 5.**
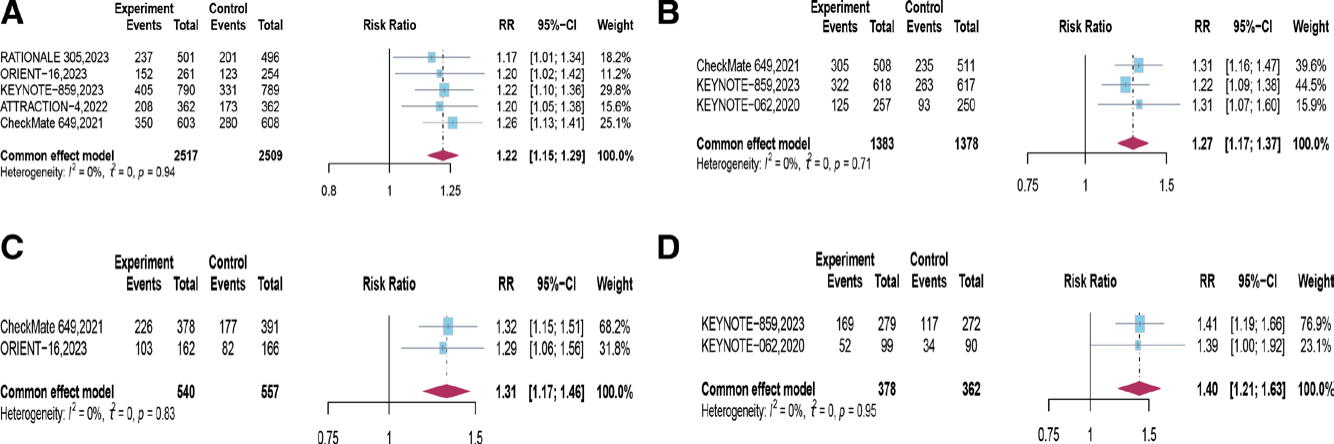
Forest plots of risk ratios (RR) comparing overall response rate (ORR) between PD-1 inhibitor combined with chemotherapy and chemotherapy based on (A) Intention-to-Treat (ITT), (B) PD-L1 combined positive score (CPS) ≥ 1, (C) CPS ≥ 5, and (D) CPS ≥ 10. CI = confidence intervals, CPS = combined positive score, ORR = overall response rate, PD-1 = programmed cell death protein 1, PD-L1 = programmed death-ligand 1, RR = risk ratios, ITT = intention-to-treat.

Four trials examined the short-term efficacy of PD-1 inhibitor combined with chemotherapy in the intended population, revealing that the PD-1 inhibitor combined with chemotherapy group improved patients’ ORR (*RR* = 1.22, 95% CI [1.15, 1.29], *P* < .00001; Fig. 5A). Subgroup analysis based on PD-L1 expression in this study demonstrated that when CPS ≥ 1 (*RR* = 1.27, 95% CI [1.17, 1.37], *P* < .00001), CPS ≥ 5 (*RR* = 1.31, 95% CI [1.17,1.46], *P* < .00001), and CPS ≥ 10 (*RR* = 1.40, 95% CI [1.21, 1.63], *P* < .00001), the PD-1 inhibitor combined with chemotherapy regimen improved the ORR for patients, with greater improvements observed with higher CPS scores (Fig. 5B–D, Table 3).

**Figure 6.**
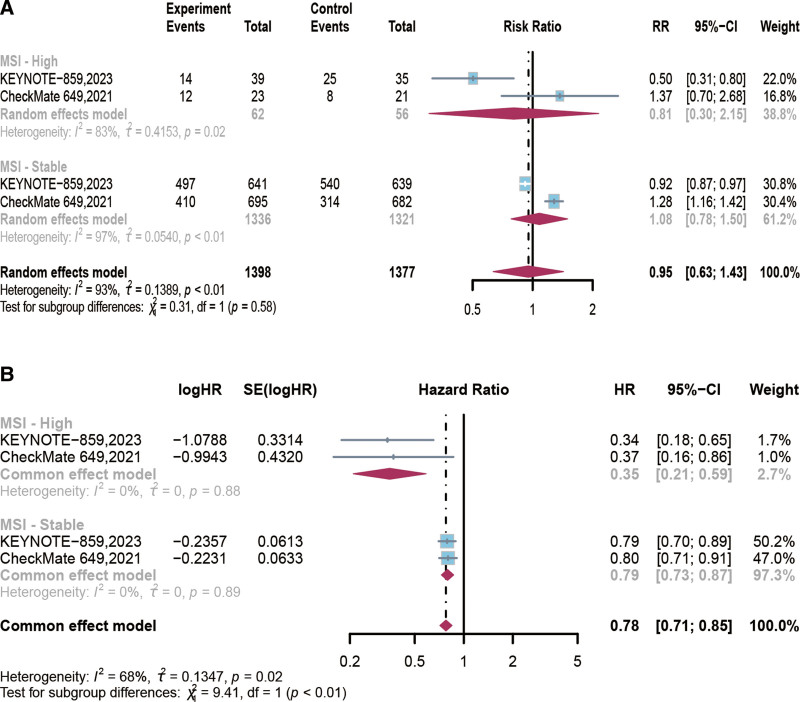
Forest plot of risk ratios (RR) for overall response rate (ORR) based on microsatellite instability (MSI) status comparing PD-1 inhibitors in combination with chemotherapy versus chemotherapy (A), Forest plot of hazard ratios (HRs) for overall survival (OS) based on microsatellite instability (MSI) status comparing PD-1 inhibitors in combination with chemotherapy versus chemotherapy (B). CI = confidence intervals, HRs = hazard ratios, MSI = microsatellite instability, ORR = overall response rate, OS = overall survival, PD-1 = programmed cell death protein 1, RR = risk ratios.

**Figure 7.**
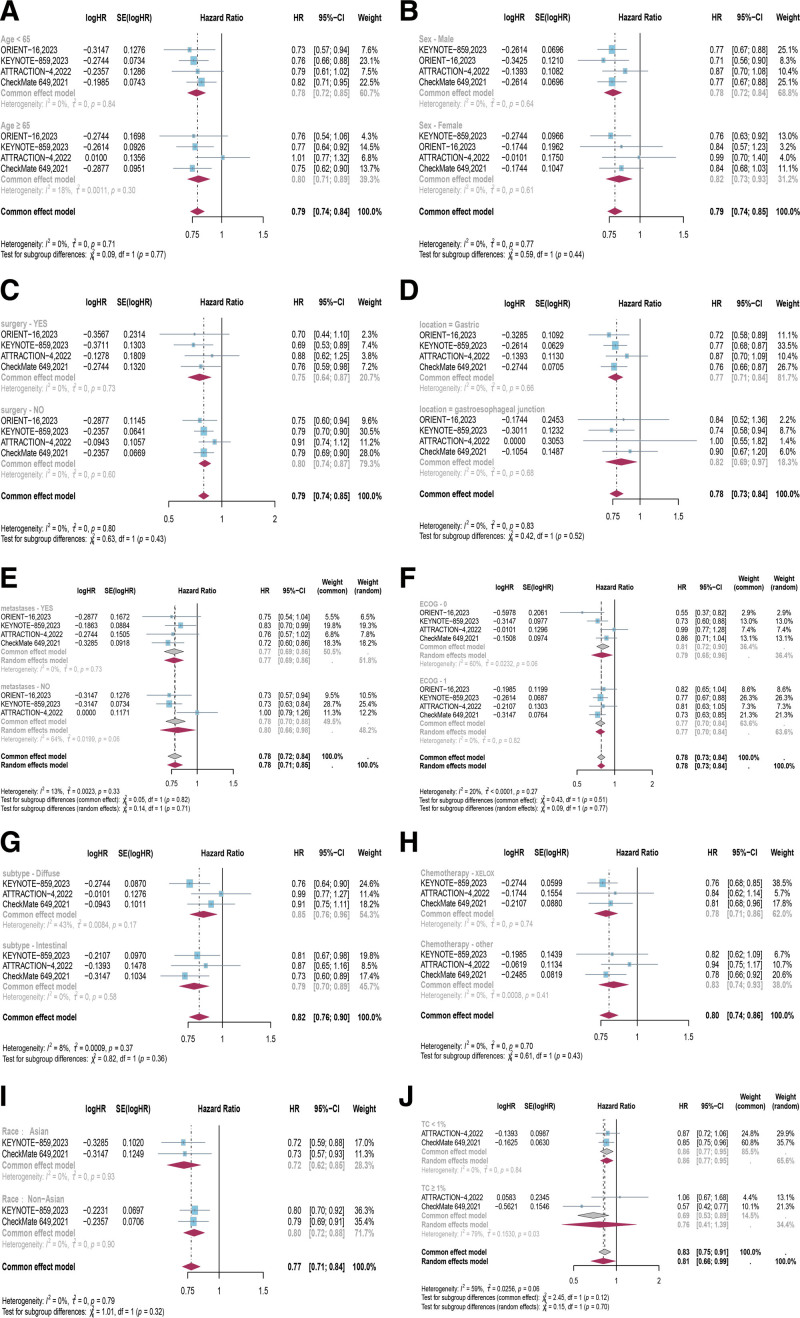
Forest plots of hazard ratios (HRs) comparing overall survival (OS) between PD-1 inhibitors in combination with chemotherapy versus chemotherapy based on the following factors: (A) age, (B) gender, (C) previous surgery, (D) primary location, (E) liver metastases, (F) ECOG PS score, (G) histological subtype, (H) chemotherapy regimen, (I) race, (J) PD-L1 expression in tumor cells. CI = confidence intervals, ECOG PS = Eastern Cooperative Oncology Group Performance Status, HRs = hazard ratios, OS = overall survival, PD-1 = programmed cell death protein 1, PD-L1 = programmed death-ligand 1.

This study, based on a subgroup analysis of MSI, showed that PD-1 inhibitors in combination with chemotherapy did not improve the ORR (*RR* = 0.81, 95% CI [0.30, 2.15], *P* = .67; Fig. 6A) in high microsatellite instability (MSI-H) patients compared with chemotherapy. However, in terms of long-term efficacy, compared to patients with microsatellite stability and their OS (*HR* = 0.79, 95% CI [0.73, 0.87], *P* < .00001), PD-1 inhibitor combined with chemotherapy significantly improved the OS for MSI-H patients (*HR* = 0.35, 95% CI [0.21, 0.59], *P* < .0001; Fig. 6B). Nevertheless, factors such as age (<65 *HR* 0.78 vs ≥ 65 *HR* 0.80; *P* = .77), gender (male *HR* 0.78 vs female *HR* 0.82; *P* = .44), previous surgery (Yes *HR* 0.75 vs No *HR* 0.80; *P* = .43), primary location (gastric *HR* 0.77 vs gastroesophageal junction *HR* 0.82; *P* = .52), race (Asian *HR* 0.72 vs Non-Asian *HR* 0.80; *P* = .32), ECOG (0 *HR* 0.79 vs 1 *HR* 0.77; *P* = .77), chemotherapy regimen (XELOX [capecitabine and oxaliplatin] *HR* 0.78 vs other *HR* 0.83; *P* = .43), histological subtype (Diffuse *HR* 0.85 vs Intestinal *HR* 0.79; *P* = .36), liver metastases (Yes *HR* 0.77 vs No *HR* 0.80; *P* = .71), and PD-L1 expression in tumor cells (<1% *HR* 0.86 vs ≥1% *HR* 0.76; *P* = .70) were unable to predict the clinical benefits of PD-1 inhibitor combined with chemotherapy compared to chemotherapy (Fig. 7, Table 4).

**Table 4:** Subgroup analysis of meta-analysis concerning overall survival.

Subgroup	Included studies	HR [95%CI]	HR (interaction action, 95% CI)	*P* (subgroups)	*I*^2^ (*P* value)	Model
Age						
<65	4	0.78 [0.72,0.85]	0.79 [0.74,0.84]	.77	0 (.71)	Common
≥65	4	0.80 [0.71,0.89]				
Sex						
Male	4	0.78 [0.72,0.84]	0.79 [0.74,0.85]	.44	0 (.77)	Common
Female	4	0.82 [0.73,0.93]				
Previous surgery						
Yes	4	0.75 [0.64,0.87]	0.79 [0.74,0.85]	.43	0 (.80)	Common
No	4	0.80 [0.74,0.87]				
Primary location						
Gastric	4	0.77 [0.71,0.84]	0.78 [0.73,0.84]	.52	0 (.83)	Common
Gastroesophageal junction	4	0.82 [0.69,0.97]				
Liver metastases						
Yes	4	0.77 [0.69,0.86]	0.78 [0.71,0.85]	.71	13 (.33)	Random
No	4	0.80 [0.66,0.98]				
ECOG PS score						
0	4	0.79 [0.65,0.96]	0.78 [0.73,0.84]	.77	20 (.27)	Random
1	4	0.77 [0.70,0.84]				
Histological subtype						
Diffuse	3	0.85 [0.76,0.96]	0.82 [0.76,0.90]	.36	8 (.37)	Common
Intestinal	3	0.79 [0.70,0.89]				
Chemotherapy regimen						
XELOX	3	0.78 [0.71,0.86]	0.80 [0.74,0.86]	.43	0 (.70)	Common
other	3	0.83 [0.74,0.93]				
Race						
Asian	2	0.72 [0.62,0.85]	0.77 [0.71,0.84]	.32	0 (.79)	Common
Non-Asian	2	0.80 [0.72,0.88]				
PD-L1 expression in tumor cells						
<1%	2	0.86 [0.77,0.95]	0.81 [0.66,0.99]	.70	59 (.06)	Random
≥1%	2	0.76 [0.41,1.39]				
MSI						
High	2	0.35 [0.21,0.59]	0.78 [0.71,0.85]	<.01	68 (.02)	Random
Stable	2	0.79 [0.73,0.87]				

ECOG PS = Eastern Cooperative Oncology Group Performance Status, MSI = microsatellite instability, PD-L1 = programmed death-ligand 1, XELOX = capecitabine and oxaliplatin .

## 5. Safety

**Figure 8.**
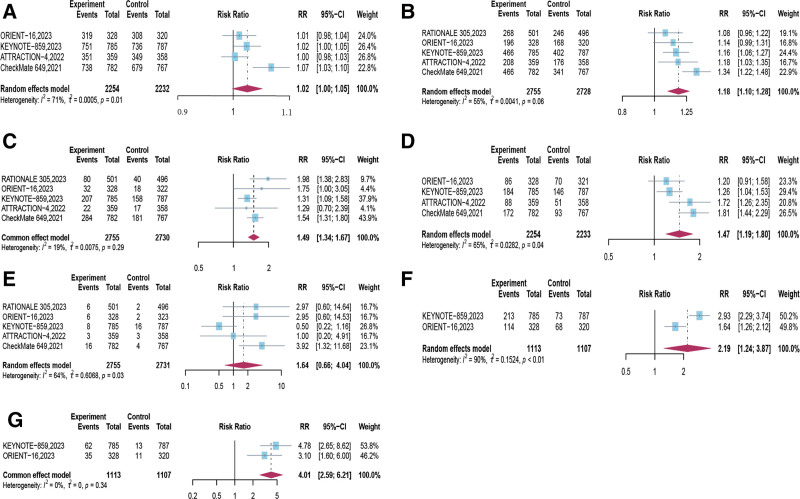
Forest plots depicting the risk ratios comparing treatment-associated adverse events (TRAEs) and immune-related adverse events (irAEs) among patients receiving PD-1 inhibitors combined with chemotherapy versus chemotherapy alone. TRAEs are categorized as any grade AEs (A), grade ≥3 (B) leading to treatment discontinuation (C), serious adverse events (D), and events leading to death (E), immune-related adverse events are categorized as any grade AEs (F) and grade ≥3 (G). AE = adverse event, CI = confidence intervals, irAEs = immune-related adverse events, PD-1 = programmed cell death protein 1, RR = risk ratios, TRAEs = treatment-associated adverse events.

In response to the study reporting safety-related data, our summarized and analyzed results indicate that treatment-related adverse events (TRAEs) were generally tolerable, encompassing any-grade adverse events (*RR* = 1.02, 95% CI [1.00, 1.05], *P* = .656; Fig. 8A), grade 3 or higher adverse events (*RR* = 1.18, 95% CI [1.10, 1.28], *P* < .0001; Fig. 8B), adverse events leading to treatment discontinuation (*RR* = 1.49, 95% CI [1.34, 1.67], *P* < .0001; Fig. 8C), severe adverse events (*RR* = 1.47, 95% CI [1.19, 1.80], *P* = .0003; Fig. 8D), and adverse events resulting in death (*RR* = 1.64, 95% CI [0.66, 4.06], *P* = .2837; Fig. 8E). Importantly, there were no statistically significant differences between the 2 treatment groups in terms of any-grade adverse events (*P* = .656) and adverse events leading to death (*P* = .2837). Furthermore, PD-1 inhibitor combined with chemotherapy increased the incidence of irAEs, including any-grade adverse events (*RR* = 2.19, 95% CI [1.24, 3.87], *P* = .007; Fig. 8F) and grade 3 or higher severe adverse events (*RR* = 4.01, 95% CI [2.59, 6.21], *P* < .001; Fig. 8G, Table 3).

## 6. Discussion

In recent years, as mid- and late-stage research outcomes of large-scale clinical trials related to GC/GEJC are published, many researchers have conducted meta-analyses based on large RCT results. Huo et al published a meta-analysis on the efficacy of ICIs in GC/GEJC, revealing a significant OS benefit of PD-1/PD-L1 inhibitors over chemotherapy alone, particularly in patients under 65 or males, although no improvement in PFS was observed.^[14]^ Cai et al included first-line phase III clinical trials of ICI combined with chemotherapy for advanced gastric adenocarcinoma or GEJ adenocarcinoma in a network meta-analysis, uncovering significant OS benefits from the immunotherapy combination, especially in patients with a CPS of 10 or higher or those with high MSI-H, indicating a statistical difference in clinical benefit.^[15]^ However, this analysis lacked subgroup analysis and did not differentiate between first-line and second-line treatments specifically. Fei et al’s meta-analysis demonstrated superior clinical efficacy of PD-1 inhibitor combined with chemotherapy as a first-line treatment over chemotherapy alone, particularly in patients with high CPS, but did not analyze safety.^[16]^ Maryam Noori et al’s meta-analysis found better clinical benefits in males with the immunotherapy combination, though with poorer safety.^[17]^ Our study specifically conducts a meta-analysis of Phase III clinical trials on the first-line treatment of patients with HER2-negative advanced GC/GEJC using PD-1 inhibitors combined with chemotherapy, systematically analyzing clinical efficacy and safety. Through this research, we aim to identify the optimal patient population for this first-line treatment approach in HER2-negative advanced GC/GEJC and to uncover other prognostic biomarkers, thereby refining and enhancing previous meta-analytical results to support clinical decision-making and treatment in this domain. To our knowledge, our meta-analysis includes the latest and highest quality RCTs related to this topic, thoroughly analyzing efficacy and safety and conducting subgroup analysis based on various characteristics to offer guidance for the application of PD-1 inhibitor combined with chemotherapy in treating patients with HER2-negative advanced GC/GEJC.

Our research findings indicate that the first-line treatment with PD-1 inhibitors combined with chemotherapy significantly enhances the short-term efficacy and long-term survival benefits in patients with HER2-negative advanced GC/GEJC, aligning with the results from the KEYNOTE-859 and CheckMate-649 trials. This improvement may be attributed to the synergistic antitumor effects achieved by enhancing tumor cell sensitivity to PD-1 inhibitors through chemotherapy and counteracting the negative immunoregulatory effects induced by chemotherapy.^[18]^ PD-1 inhibitors in combination with chemotherapy were more effective in patients with CPS ≥ 10 and MSI-H. This is consistent with current clinical guidelines and further illustrates its status as a class I recommended treatment option. The expression levels of PD-L1 CPS and MSI have the potential to serve as a biomarker for HER2-negative advanced GC/GEJC. Currently, CPS is commonly used in clinical guidelines to define PD-L1 expression levels in tumor patients, where higher CPS scores correlate with increased effectiveness of PD-1 inhibitor treatment. This is likely due to the ability of PD-1 inhibitors to prevent the binding of PD-L1 to PD-1 receptors on activated T cells, allowing the immune system to target and attack the tumor cells. Furthermore, studies have reported better immunotherapy prognosis in MSI-H GC patients, likely due to the high infiltration of CD8^+^ T cells, indicating a greater sensitivity to immunotherapy.^[19]^ However, our study found that characteristics such as age, gender, previous surgery, primary location, liver metastases, ECOG performance status score, histological subtype, chemotherapy regimen, race, and PD-L1 expression in tumor cells did not predict clinical efficacy. In terms of safety, TRAEs were generally tolerable, although the combination of PD-1 inhibitor and chemotherapy resulted in a higher incidence of immune-mediated adverse events.

The expression level of PD-L1 is considered a potential biomarker for predicting the efficacy of immunotherapy; however, the optimal threshold for its expression has yet to be defined. PD-L1 expression is commonly assessed using CPS, TPS, and IPS scores. Despite the European Medicines Agency and National Comprehensive Cancer Network guidelines incorporating CPS into their recommendations and making targeted treatment suggestions, the FDA-approved population remains broad, leaving the differential role of PD-L1 expression in GC unclear. Our study predominantly evaluated PD-L1 expression using CPS in the included RCTs. Research suggests that the efficacy of PD-1 inhibitors in GC patients is associated with PD-L1 expression levels.^[20]^ Tumor cells, tumor-associated immune cells, and stromal cells often express the ligand PD-L1 to evade immune attack. PD-1 inhibitors can disrupt this interaction between PD-L1 and PD-1 receptors on activated T cells, enabling the immune system to target and eliminate tumor cells. Consistent with our findings, we observed benefits in OS, PFS, and ORR among patients with CPS scores of ≥1, ≥5, and ≥10 when treated with the first-line combination of PD-1 inhibitors and chemotherapy, with higher PD-L1 expression levels correlating with better therapeutic outcomes. Therefore, considering the patient’s overall condition, the first-line treatment with PD-1 inhibitors combined with chemotherapy appears to offer greater clinical efficacy in patients with CPS ≥ 10 in HER2-negative advanced GC/GEJC.

Immunotherapy, particularly with ICIs, has demonstrated enhanced clinical efficacy in solid tumors characterized by a high tumor mutational burden.^[21]^ Our research findings similarly reveal that PD-1 inhibitors in combination with chemotherapy significantly improved the OS in MSI-H patients, with a notable difference between groups (*P* < .01). The increased expression of neoantigens in tumors with MSI-H or mismatch repair deficiencies, often associated with higher mutation rates and methylation,^[21]^ may explain their enhanced sensitivity to immunotherapy due to the substantial infiltration of CD8^+^ T cells in MSI-H tumors. While MSI-H patients showed a better ORR, no significant difference was observed between groups (*P* = .58), possibly related to the shorter clinical observation period. It is our belief that patients with MSI-H may benefit more from combination therapy, though further trials are needed to confirm this and assess the safety of combined medication use.

Studies such as those by Kugel et al suggest that older populations may experience greater clinical benefits from ICIs, with Wu et al reporting varying mechanisms across different age groups.^[22,23]^ Moreover, research indicates that gender differences can impact innate and adaptive immune responses, potentially leading to variations in clinical efficacy of ICI treatment between male and female patients.^[24]^ However, our study found no significant clinical difference in efficacy between PD-1 inhibitor combined with chemotherapy versus chemotherapy alone when age was divided at 65 years, nor did we observe efficacy differences between genders in the treatment groups. Our meta-analysis results contradict those by Huo et al and Maryam Noori et al, possibly related to the toxic effects of combined drug use, though the reasons remain unclear. The role of age and gender in the efficacy of immunotherapy continues to be a topic of debate, requiring further confirmation in future large-scale clinical trials.

Regarding safety, TRAEs were generally tolerable; however, the incidence of irAEs was higher in the PD-1 inhibitor combined with chemotherapy group, including hypothyroidism, hyperthyroidism, pneumonitis, colitis, thyroiditis, adrenal insufficiency, hepatitis, severe skin reactions, increased amylase, rash, etc. The activation of the immune system by ICIs can lead to an increased occurrence of inflammation or irAEs. When combining drugs, the interaction between drugs and their distinct targets must be considered, taking into account the patient’s baseline state and potential adverse reactions of selected drugs to choose a treatment plan that is both effective and safe.

This study has limitations. The included studies in our meta-analysis vary in sample size and follow-up duration, introducing inherent biases that may be amplified after meta-analysis. Moreover, in different trials, the dosage of the same treatment regimen might vary, but for indirect comparisons of different treatment efficacies and safety, minor differences are often overlooked. In subgroup analysis, due to the small number of individuals analyzed, results should be interpreted with caution. Lastly, as this is a literature-based meta-analysis rather than an individual patient data meta-analysis, it may be subject to publication bias. Hence, further verification studies in patients receiving first-line treatment with PD-1 inhibitors combined with chemotherapy for HER2-negative advanced GC/GEJC are crucial.

In summary, through meta-analysis, our study affirms the clinical benefits of the first-line treatment strategy of PD-1 inhibitors combined with chemotherapy in the treatment of HER2-negative advanced GC/GEJC. Compared to chemotherapy alone, the first-line treatment with PD-1 inhibitors combined with chemotherapy significantly prolongs the OS, PFS, and improves the ORR in patients with HER2-negative advanced GC/GEJC. Patients with high CPS or MSI-H, after considering drug safety and patient baseline conditions, may be prioritized for the first-line treatment approach with PD-1 inhibitors combined with chemotherapy, with higher CPS scores correlating with greater therapeutic benefits. However, current research on the first-line treatment with PD-1 inhibitors combined with chemotherapy for HER2-negative advanced GC/GEJC is limited, necessitating further high-quality trial research and longer clinical practice to confirm and monitor effectiveness, safety, and applicable populations. In addition, more and more precise biomarkers still need to be tapped.

## Author contributions

**Conceptualization and design:** Yu Wei, Muhetaibaier Hairoula, Li Zhang.

**Search strategy, selection criteria, and search literature:** Xiaoli Ma, Leiyu Cao, Yan Gao.

**Collection and assembly of data:** Chengcheng Qu, Kalima Muhetaer, Wen Yi

**Statistical analysis and visualization:** Muhetaibaier Hairoula

**Writing – original draft:** Muhetaibaier Hairoula, Yu Wei.

**Writing – review & editing:** Muhetaibaier Hairoula, Yu Wei, Li Zhang.
